# The deubiquitinase USP15 modulates cellular redox and is a therapeutic target in acute myeloid leukemia

**DOI:** 10.1038/s41375-021-01394-z

**Published:** 2021-08-31

**Authors:** Madeline Niederkorn, Chiharu Ishikawa, Kathleen M. Hueneman, James Bartram, Emily Stepanchick, Joshua R. Bennett, Ashley E. Culver-Cochran, Lyndsey C. Bolanos, Emma Uible, Kwangmin Choi, Mark Wunderlich, John P. Perentesis, Timothy M. Chlon, Marie-Dominique Filippi, Daniel T. Starczynowski

**Affiliations:** 1 Division of Experimental Hematology and Cancer Biology, Cincinnati Children’s Hospital Medical Center, Cincinnati, OH USA; 2 Department of Cancer Biology, University of Cincinnati College of Medicine, Cincinnati, OH USA; 3 Division of Oncology, Cincinnati Children’s Hospital Medical Center, Cincinnati, OH USA; 4 Department of Pediatrics, University of Cincinnati College of Medicine, Cincinnati, OH USA

**Keywords:** Acute myeloid leukaemia, Cancer therapy

## Abstract

Ubiquitin-specific peptidase 15 (USP15) is a deubiquitinating enzyme implicated in critical cellular and oncogenic processes. We report that USP15 mRNA and protein are overexpressed in human acute myeloid leukemia (AML) as compared to normal hematopoietic progenitor cells. This high expression of USP15 in AML correlates with KEAP1 protein and suppression of NRF2. Knockdown or deletion of USP15 in human and mouse AML models significantly impairs leukemic progenitor function and viability and de-represses an antioxidant response through the KEAP1-NRF2 axis. Inhibition of USP15 and subsequent activation of NRF2 leads to redox perturbations in AML cells, coincident with impaired leukemic cell function. In contrast, USP15 is dispensable for human and mouse normal hematopoietic cells in vitro and in vivo. A preclinical small-molecule inhibitor of USP15 induced the KEAP1-NRF2 axis and impaired AML cell function, suggesting that targeting USP15 catalytic function can suppress AML. Based on these findings, we report that USP15 drives AML cell function, in part, by suppressing a critical oxidative stress sensor mechanism and permitting an aberrant redox state. Furthermore, we postulate that inhibition of USP15 activity with small molecule inhibitors will selectively impair leukemic progenitor cells by re-engaging homeostatic redox responses while sparing normal hematopoiesis.

## Introduction

Acute myeloid leukemia (AML) is a hematopoietic malignancy defined by ineffective hematopoiesis, defects in myeloid differentiation, and an accumulation of myeloid blasts in the bone marrow (BM) and peripheral blood (PB) of patients. The only curative therapy for AML is allogeneic stem cell transplantation, for which a number of patients are ineligible, and many patients will relapse despite this treatment [1]. The response of leukemic hematopoietic stem and progenitor cells (HSPCs) to these therapies is dependent on coinciding mutations, cytogenetic alterations, and adaptive resistance mechanisms [2]. RNA-sequencing and genome sequencing of AML samples have uncovered relevant genetic and transcriptional mechanisms underlying the pathogenesis of AML [2]. However, adaptive mechanisms reliant on cellular signaling and post-translational modifications play substantial roles in maintaining oncogenic cell states, and comparatively, these are not thoroughly understood functions in leukemic cells.

Similar to proteins, metabolites in HSPCs can act as secondary messengers to inform cell function and fate decisions under nutrient deprivation, oxidative stress, and cell growth [3–5]. Generated as a biproduct of mitochondrial oxidative phosphorylation, reactive oxygen species (ROS) are unsurprisingly elevated in rapidly proliferating leukemia cells which have high metabolic demands. While excess ROS is categorically DNA-damaging and compromises the integrity of normal HSCs [6], basal levels of ROS are required for stem cell function. However, leukemia cells persist despite elevated levels of ROS, likely by upregulating compensatory pathways [7]. Moreover, transcription factors such as FOXOs, NRF2, NF-κB, and HIF1-α are regulated by ROS, as are glycolytic enzymes, mitochondrial transporters, p38-MAPK, and mTOR pathways [8–12]. ROS and metabolic processes are intricately intertwined in hematopoiesis and leukemic cells leverage these mechanisms to drive cell survival programs despite oxidative stress. Importantly, this co-opted network of malignant hematopoietic cell metabolism and redox homeostasis represents a target-rich space for selectively depleting leukemic cells [13].

Ubiquitination is a rapid, reversible, and dynamic post-translational modification implicated in protein degradation, activation of signaling pathways, translational regulatory mechanisms, chromatin remodeling, and redox homeostasis [14–18]. Enzymes that modify ubiquitin, such as E3 ligases or deubiquitinating enzymes (DUBs), are critical to these processes. DUBs have emerged as a promising new class of therapeutic targets in a number of cancers, including but not limited to prostate cancer [19], melanoma [20], breast cancer [21], multiple myeloma [22], neuroblastoma [23], and AML [24, 25]. Therefore, understanding the underlying cell biology of DUBs and their functional roles in transformed cells versus normal cells is valuable to identify potential targets and inform drug discovery efforts. To that end, we find the DUB USP15 to be highly expressed in AML. USP15 belongs to a class of ubiquitin-specific proteases (USPs) and it is most commonly reported to disrupt proteasomal degradation of its substrates by removing lysine (K) 48 linked ubiquitin chains or impairing signaling scaffolds by removing K63 linked ubiquitin chains. Our previous study in hematopoietic cells uncovered a TIFAB-USP15 complex that affects p53 signaling and cellular stress responses [26, 27], indicating that USP15 might have several important roles in leukemic progenitors.

In light of the well-documented role of oxidative cell states in AML, the need to understand the molecular mechanisms underlying redox sensing is becoming increasingly important for the purpose of identifying tractable therapeutic targets. Several reports indicate that a key substrate of USP15 is a critical redox sensor, KEAP1 [28, 29]. KEAP1 is an E3 cullin-RING ligase adapter harboring multiple cysteine residues that, when exposed to excess ROS, form intramolecular disulfide bonds that render KEAP1 inactive [30]. Under oxidative stress, KEAP1 is restricted by ubiquitin/proteosome-mediated degradation, thus liberating the transcription factor NRF2 from proteasomal degradation and cytoplasmic sequestration [30, 31]. Specifically, NRF2 controls antioxidant transcriptional programs by recognizing ARE-motif-containing genes. In normal hematopoietic cells, deletion of USP15 decreased the levels of KEAP1 [26], indicating the potential for USP15 to modify redox sensing in normal and malignant hematopoiesis. USP15 is also reported to repress p53 and mediate DNA-damage responses in hematopoietic cells [32], which may be related to the oxidative stress response in leukemia [33]. However, the cellular and functional requirements of USP15 in AML are not fully understood. We hypothesize that high levels of USP15, as observed in AML, are important for leukemic progenitor function, yet dispensable for normal hematopoietic cells, due to their increased reliance on aberrant redox states. Through genetic and pharmacologic approaches, our studies delineate the requirement of USP15 in AML through its rewiring of oxidative stress sensing and reveal the tractability of USP15 as a potential therapeutic target in AML.

## Results

### AML exhibits high USP15 expression compared to other tumor types and normal hematopoietic cells

**Fig. 1:**
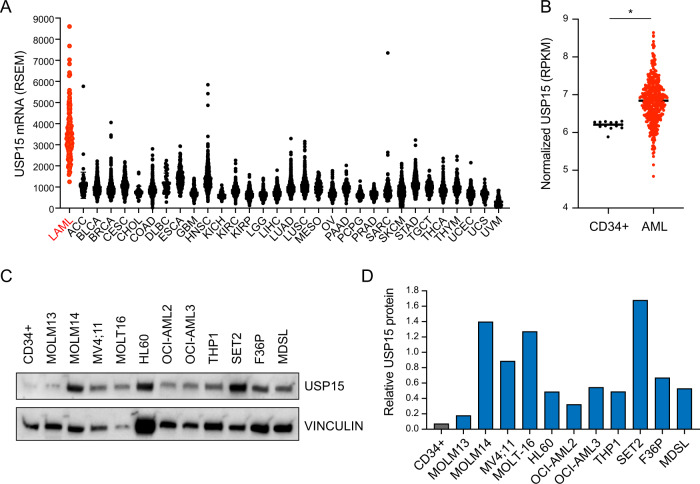
AML exhibits high USP15 expression compared to other tumor types and normal hematopoietic cells. **A** RSEM expression values for USP15 mRNA in the TCGA PanCancer study. LAML in red = acute myeloid leukemia. **B** Normalized RPKM values for USP15 RNA expression in healthy control CD34+ samples and AML patient samples from the BEAT-AML dataset. *P < 0.05. **C** Immunoblot for USP15 and vinculin (loading control) on lysates generated from healthy CD34+ control cells and the indicated human AML cell lines. Vinculin expression was variable among the different cell types. Shown are representative data from 2 replicate experiments. **D** Relative expression of USP15 protein expression (normalized to VINCULIN) was determined by densitometry performed in ImageJ software. Shown are the normalized values from panel (**C**).

Previously we reported that the del(5q) MDS gene TIFAB controls HSPC functions under stressed conditions through direct interaction and regulation of USP15 function [26, 27, 34, 35]. The relevance of USP15 in del(5q) MDS prompted us to examine the broader role of USP15 in cancers. Utilizing publicly available data from The Cancer Genome Atlas, we first evaluated USP15 mRNA expression across all reported tumor types. Although USP15 transcripts are readily expressed within many tumor types, their expression is remarkably higher in AML samples (Fig. 1A). We reasoned that this observation could either be due to generally high USP15 expression in myeloid tissues compared to other tissue types or due to a unique upregulation of USP15 in leukemia versus normal hematopoietic cells. To address this possibility, we analyzed USP15 RNA expression in AML patient samples (*n* = 451) as well as healthy CD34+ control samples (*n* = 13) [36]. We observe that, compared to normal CD34+ HSPCs, the patient samples expressed significantly higher levels of USP15 transcripts (*P* < 0.0001) (Fig. 1B). Within normal hematopoietic progenitor cell subtypes, USP15 RNA expression is lower in lineage-committed granulocyte-monocyte progenitors (GMP) than in HSC, multipotent progenitors (MPP), or common myeloid progenitors (CMP) (Fig. S1A). To independently corroborate these observations, we assembled a panel of human leukemic cell lines and performed qPCR to evaluate USP15 mRNA expression compared to normal CD34+ cells. Compared to the healthy controls, all of the AML cell lines (HL60, MDSL, OCI-AML2, OCI-AML3, and SET-2) exhibit increased USP15 mRNA (Fig. S1B). To determine whether the change in USP15 transcripts results in an increase in USP15 protein, we performed immunoblotting for USP15 in CD34+ cells and a panel of leukemia cell lines (Fig. 1C). USP15 protein is highest in MOLM14, MV4;11, MOLT16, and SET2 (>8-fold), while USP15 protein was moderately overexpressed in HL60, OCI-AMl2, OCI-AML3, THP1, F36P, and MDSL (>2-fold) as compared to normal CD34+ cells (Fig. 1C, D). Our analyses indicate that USP15 expression in AML exceeds that of other cancer types and that AML patient samples and cell lines express significantly higher levels of USP15 as compared to normal hematopoietic cells.

### Knockdown of USP15 impairs human leukemic cell function in vitro and in vivo

**Fig. 2:**
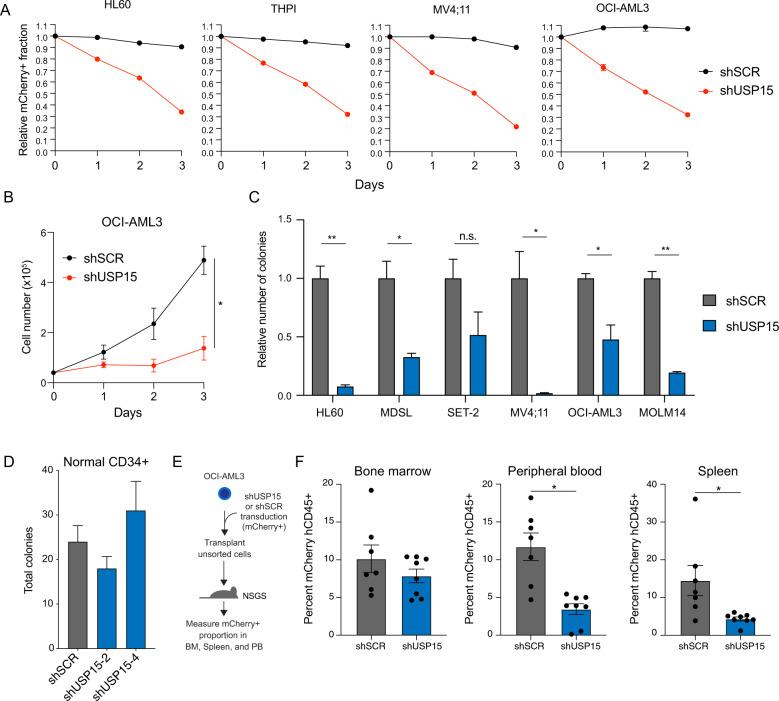
Knockdown of USP15 impairs human leukemic cell function in vitro and in vivo. **A** Relative proportion of the indicated AML cell lines transduced with shRNAs targeting USP15 (shUSP15) or a scrambled control shRNA (shSCR) co-expressing mCherry. The percent of mCherry+ cells was initially determined by flow cytometry at 48 h after transduction (Day 0). The proportion of mCherry+ cells was normalized to the percent mCherry+ on Day 0 for each group. Data reflects two independent assays (*n* = 3 per group). **B** Total cell number of OCI-AML3 cells transduced with shRNAs targeting USP15 (shUSP15) or a scrambled control shRNA (shSCR). *P < 0.05. Data reflects two independent assays (*n* = 3 per group). **C** The relative number of colonies formed in methylcellulose from the indicated AML cells transduced with shRNA targeting USP15 (shUSP15) or a scrambled control shRNA (shSCR) co-expressing mCherry. Cells were sorted for mCherry expression prior to plating. *P < 0.05; **P < 0.005; ns, not significant. (*n* = 3) Data reflects two independent assays. **D** The total number of colonies formed in methylcellulose from healthy CD34+ cells transduced with shRNAs targeting USP15 (shUSP15) or a scrambled control shRNA (shSCR) co-expressing mCherry (*n* = 3 per group). *P < 0.05. **E** Schematic depicting unsorted OCI-AML3 cells transduced with shRNAs targeting USP15 (shUSP15) or a scrambled control shRNA (shSCR) xenografted into NSGS mice. **F** Percent of human CD45+populations expressing shSCR (*n* = 7 mice per group) or shUSP15 (*n* = 8 mice per group) (mCherry+) was evaluated in the bone marrow, peripheral blood, and spleen at the time of death. *P < 0.05.

We next evaluated the requirement of USP15 for leukemic cell function across multiple AML cell types and for normal human CD34+ cells. To test whether the knockdown of USP15 impaired leukemic cell proliferation, we expressed shRNAs targeting USP15 (shUSP15) in HL60, MV4:11, THPI, and OCI-AML3 using lentiviral vectors co-expressing a mCherry reporter or scrambled control shRNA (shSCR) (Fig. S1C). After viral transduction of the AML cell lines, we tracked the mCherry+ population (relative to non-transduced mCherry− cells) in vitro by flow cytometry for up to 3 days. The proportion of mCherry+ AML cells in the population expressing the shRNA control persisted over 3 days, while the proportion of mCherry+ AML cells expressing shUSP15 significantly reduced over this time course (Fig. 2A). To evaluate the effects of USP15 depletion on AML cell proliferation and viability, we selected OCI-AML3 for further examination. Transduced OCI-AML3 cells expressing shRNAs targeting USP15 (shUSP15) or shSCR were isolated by flow cytometry (mCherry+) and then immediately cultured in vitro. Knockdown of USP15 significantly impaired the growth of viable (Trypan blue exclusion) OCI-AML3 cells as compared to shSCR-expressing cells (Fig. 2B). In parallel, we expressed shRNAs targeting USP15 (shUSP15) or shSCR in the panel of AML cell lines and then sorted mCherry+ cells were plated in methylcellulose to measure the cell-intrinsic effects of USP15 knockdown on leukemic progenitor function. Knockdown of USP15 impaired the colony-forming ability of the AML cell lines as compared to AML cells expressing shSCR (Fig. 2C). In contrast, knockdown of USP15 did not have observable effects on normal human CD34+ colony-forming ability (Fig. 2D). To determine the effects of USP15 knockdown on OCI-AML3 cells in vivo, we also transplanted 5 × 10^5^ cells from an unsorted population of OCI-AML3 cells transduced with lentiviral vectors co-expressing the mCherry reporter and shUSP15 or shSCR (51–46% mCherry+, respectively) into NOD-scid IL2Rγnull-mice expressing human IL3, GM-CSF (CSF2) and SCF (KITLG) (NSGS) (Fig. 2E). As the mice succumbed to leukemia, we evaluated the percent of mCherry+ AML cells in the human CD45+ fraction of cells from the blood, spleen, and BM. The percent contribution of the mCherry+ AML cells to the human CD45 fraction was significantly reduced in the blood and spleen of the groups that received the shUSP15-mixed population as compared to the shSCR group, despite having comparable representation at the start of the transplant (Figs. 2F and S1C). However, we observed only a modest reduction in OCI-AML3 cells expressing shUSP15 (mCherry+ fraction) in the BM. Collectively, these data indicate that loss of USP15 impairs AML cells in vitro and in vivo by limiting the rate of cell growth and restricting leukemic progenitor function.

### Deletion of USP15 impairs murine myeloid leukemia in vivo

**Fig. 3:**
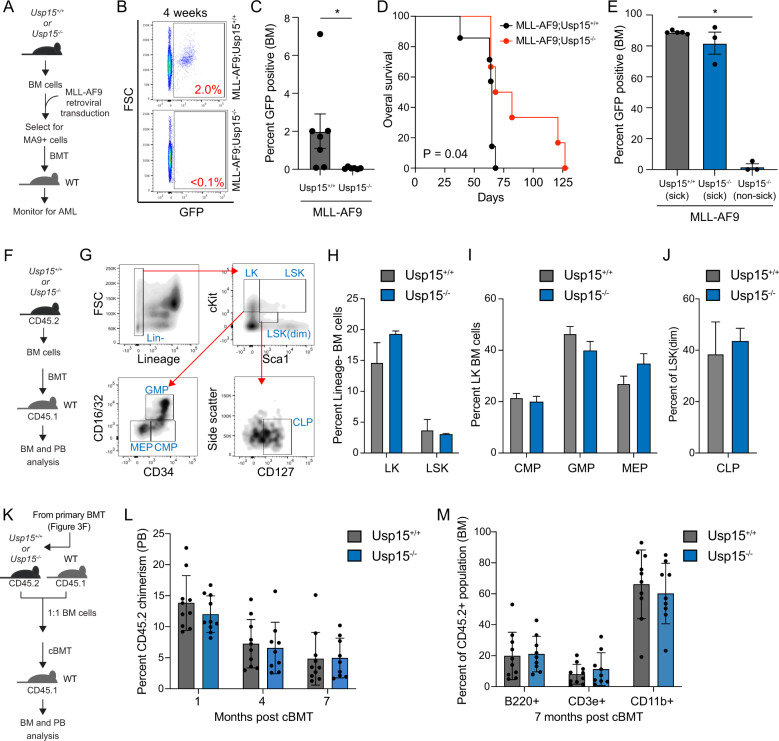
Deletion of USP15 impairs murine AML in vivo but is tolerated during normal hematopoiesis. **A** Schematic overview of the generation of a murine model of MLL-AF9 myeloid leukemia using Usp15−/− and Usp15+/+ BM cells. BM cells were transduced with retrovirus encoding MLL-AF9 (MA9) and GFP. After the selection of GFP-expressing cells, lethally-irradiated mice were engrafted with the AML cells and then monitored for engraftment and overall survival. **B** Representative analysis of the leukemic cell burden in the BM of mice four weeks after engraftment with MLL-AF9;Usp15+/+ and MLL-AF9;Usp15−/− AML cells. **C** Summary of the leukemic cell burden in the BM of mice four weeks after engraftment with MLL-AF9;Usp15+/+ (*n* = 7 mice per group) and MLL-AF9;Usp15−/− (*n* = 6 mice per group) AML cells. *P < 0.05. **D** Kaplan-Meier analysis of overall survival of mice engrafted with MLL-AF9;Usp15+/+ (*n* = 7) and MLL-AF9;Usp15−/− (*n* = 6) AML cells. *P = 0.04. **E** Summary of the leukemic cell burden in the BM of mice at the time of death (“sick”) at ~10 weeks after engraftment with MLL-AF9;Usp15+/+ and MLL-AF9;Usp15−/− AML cells and remaining mice (“non-sick”) at 10 weeks. **F** Schematic overview of the generation of BM engraftment of Usp15−/− and Usp15+/+ BM cells into lethally-irradiated recipient mice. BM cells (CD45.2) from Usp15−/− and Usp15+/+ mice were engrafted into lethally-irradiated mice (CD45.1) and then BM and PB were analyzed by immunophenotyping. **G** Representative flow plots of the gating strategy for hematopoietic progenitor populations in the BM of recipient mice, including Lineage-negative (Lin−), Lineage-c-Kit+ (LK), Lineage-c-Kit+Sca1+ (LSK), granulocyte-monocyte progenitors (GMP), megakaryocyte-erythroid progenitors (MEP), and common myeloid progenitors (CMP), and lymphoid progenitors (CLP). **H**–**J** Frequency of LK, LSK, CMP, GMP, and MEPs populations from the BM of recipient mice transplanted with Usp15+/+ and Usp15−/− BM cells. (*n* = 5 mice per group). **K** Schematic overview of the generation of competitive BM chimeras using Usp15−/− and Usp15+/+ BM cells. BM cells (CD45.2) from Usp15−/− and Usp15+/+ mice and wild-type (WT) BM cells (CD45.1) were mixed at equal proportions and then engrafted into lethally-irradiated mice (CD45.1). Percent of CD45.2+ cells in the PB and BM was measured at the indicated time points. **L** Peripheral blood chimerism of cBMT recipients at 1, 4, and 7 months post-transplant (*n* = 10 mice per group). **M** Frequency of Usp1+/+ and Usp15−/− lineage-committed B-cell (B220), T-cell (CD3e), and myeloid (CD11b) progenitors in the bone marrow at 7-months post-cBMT (*n* = 10 mice per group).

To further evaluate the relevance of USP15 in AML, Lin- BM cells isolated from Usp15+/+ and Usp15−/− mice were retrovirally transduced with vectors expressing MLL-AF9 (MSCV-IRES-GFP) (Fig. 3A). The retroviral MLL-AF9 model generates a rapid and fully penetrant AML [37]. We employed our USP15 knockout mouse model that results in complete loss of USP15 protein [26]. Transduced Lin- BM cells were first enriched for leukemic cells by serial replating in methylcellulose (Fig. 3A). By the third plating, we confirmed that nearly all cells express MLL-AF9 (>95%). As expected, we observed a reduction in the colony-forming ability of the MLL-AF9;Usp15−/− AML cells as compared to MLL-AF9;Usp15+/+ AML cells (Fig. S2A). Equal numbers of Usp15+/+;MLL-AF9 and Usp15−/−:MLL-AF9 AML cells were then transplanted (along with BM mononuclear helper cells) into lethally-irradiated BoyJ recipient mice. BM aspirates four weeks post engraftment revealed that mice transplanted with Usp15−/−;MLL-AF9 cells exhibited reduced leukemic burden in the BM as compared to mice transplanted with Usp15+/+;MLL-AF9 cells (Fig. 3B, C). Mice engrafted with Usp15+/+;MLL-AF9 cells developed leukemia with a median survival of 70 days (Fig. 3D). By 75 days, all mice engrafted with Usp15+/+;MLL-AF9 cells developed leukemia (Fig. 3D). In contrast, the mice engrafted with Usp15−/−;MLL-AF9 cells developed a significantly delayed leukemia, which prolonged the disease for several mice beyond 125 days (Fig. 3D). As expected, moribund Usp15+/+;MLL-AF9 and Usp15−/−;MLL-AF9 mice at approximately day 70 exhibited a high proportion of leukemic cells in the BM (>80% GFP+ cells), at the same point of the analysis, the remaining Usp15−/−;MLL-AF9 mice harbored <6% leukemic cells in the BM (Fig. 3E). These findings confirm that loss of USP15 can suppress development of AML in vivo.

### Deletion of USP15 does not affect normal short-term or long-term hematopoiesis

Given that deletion of USP15 impairs MLL-AF9 AML cell engraftment and delays leukemia development in vivo, we wanted to determine the consequences of USP15 deletion on normal hematopoiesis. Whole-body deletion of USP15 had no observable effects on blood cell formation nor impacted the lifespan of the mice (data not shown). To determine the effects of USP15 deletion on regenerative hematopoiesis, BM cells from wild type (Usp15+/+) or USP15-deficient (Usp15−/−) mice (C57Bl/6 CD45.2) were transplanted into lethally-irradiated CD45.1 BoyJ and then hematopoiesis was monitored in the recipient mice (Fig. 3F). Four months post-transplantation, BM cells were isolated from recipient mice (*n* = 15 per group, pooled in groups of 5 for a total of 3 replicate samples) and examined by immunophenotyping (Fig. 3G). The proportion of Lineage− (Lin−) cells was similar between mice engrafted with Usp15+/+ and Usp15−/− BM cells (data not shown). The proportion of Lin-cKit+ (LK), Lin-cKit + Sca1+ (LSK), CMP, GMP, and megakaryocyte-erythroid progenitors (MEP) were also similar between mice engrafted with Usp15+/+ and Usp15−/− BM cells 4 months post-transplantation (Fig. 3H–J) and the frequency of long-term HSCs (LT-HSCs) was comparable at 15 months post-transplant (Fig. S2B). Consistent with the lack of meaningful changes in BM HSPCs proportions between mice reconstituted with Usp15+/+ and Usp15−/− BM cells, the peripheral blood neutrophils, lymphocytes, red blood cells, and platelets were unchanged between the mice engrafted with Usp15+/+ and Usp15−/− BM cells after 1-year post-transplantation, suggesting USP15 is not required for short-term or long-term regenerative hematopoiesis (Fig. S2C). To rigorously evaluate whether intrinsic HSC function is affected upon deletion of Usp15, we performed competitive transplants utilizing BM cells from recipient mice described in Fig. 3F. Equal numbers of BM cells isolated from mice reconstituted with Usp15+/+ and Usp15−/− BM cells (CD45.2) were mixed with wild-type competitor cells (CD45.1), transplanted into lethally-irradiated congenic recipient mice, and then donor-derived (CD45.2) hematopoietic chimerism was determined in the BM and PB by fluorescence-activated cell sorting (FACS) (Fig. 3K). The hematopoietic contribution of Usp15−/− BM cells to PB chimerism was comparable to Usp15+/+ BM donor cells for up to 7 months post-transplantation (Fig. 3L). We also do not observe any significant changes in Usp15−/− BM donor cell contribution to lymphoid (B220, CD3e) and myeloid (CD11b) lineages in the BM of the recipient mice (Fig. 3M). Taken together, these data indicate that the deletion of Usp15 does not significantly impact the short-term nor long-term function of hematopoietic and mature blood cells.

### USP15 expression correlates with disrupted redox signaling in AML

**Fig. 4:**
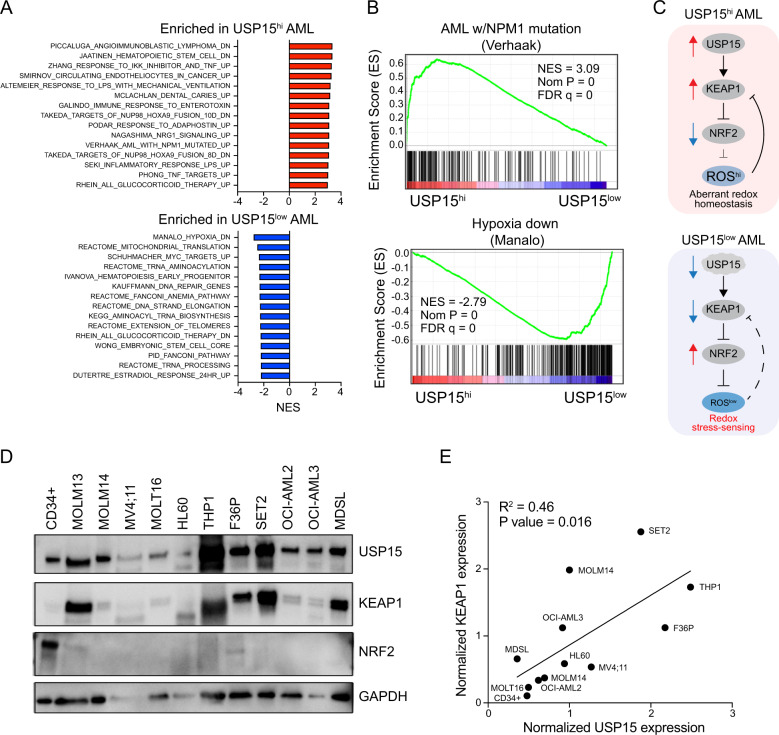
USP15 expression correlates with disrupted redox signaling in AML. **A** Normalized enrichment scores for the top 15 upregulated (red, top) and downregulated (blue, bottom), significantly altered gene sets in USP15^hi^ versus USP15^low^ AML patient samples from the BEAT-AML dataset. *P*-value < 0.05. **B** Selected gene set enrichment plots for USP15^hi^ and USP15^low^ AML groups. Shown is the enrichment of NPM1-mutated AML genes in USP15^hi^ AML and enrichment of hypoxia-related genes in USP15^low^ AML, (*P* < 0.00001). NES normalized enrichment score. **C** Schematic overview of the USP15-KEAP1-NRF2 pathway as it pertains to observations in USP15^high^ and USP15^low^ AML. Elevated USP15 is required to maintain redox homeostasis by directly sustaining KEAP1 protein expression and as a result inhibition of NRF2. In the absence of USP15, KEAP1 expression is diminished resulting in NRF2-mediated redox stress. **D** Immunoblot of USP15, KEAP1, NRF2, and GAPDH in normal CD34+ cells and a panel of AML cell lines. Representative data from 2 replicate experiments. **E** An *X*, *Y* coordinate dot plot using the quantities of USP15 and KEAP1 determined by mean gray area value in ImageJ, normalized to GAPDH for each sample from panel (**D**). Linear regression was performed to calculate the R-squared and P-values. The best-fit line is shown.

To determine the cellular pathways in AML patients that are likely to be affected by changes in USP15 expression, we stratified AML patient samples into USP15-high and USP15-low expression groups. We performed gene-set enrichment analysis to identify pathways that are significantly associated with USP15-high AML and USP15-low AML (Fig. 4A). The most negatively enriched gene-sets in USP15-high AML (enriched in USP15-low AML) included genes responding to hypoxia, mitochondrial translation, and activation by MYC (Fig. 4A, B). The most positively enriched gene-sets in USP15-high AML included hematopoietic stem cell maintenance, innate-immune, and inflammatory responses, and genes associated with NPM1-mutated AML (Fig. 4B). Based on the enrichment analyses of USP15-high AML, our previous finding linking USP15 to the hematopoietic cell stress response, and reports that the redox sensor KEAP1 is a relevant substrate regulated by USP15, we hypothesized that impinging USP15 in AML cells would modulate the KEAP1-NRF2 pathway, thus affecting cellular redox homeostasis (Fig. 4C). The deubiquitinating activity of USP15 protects KEAP1 function by preventing proteosome-mediated degradation (Fig. S3A). KEAP1 negatively regulates NRF2 by ubiquitination and cytoplasmic sequestration. Therefore, we hypothesized that low levels of USP15 would diminish KEAP1 and lead to aberrant NRF2 activation; conversely, high expression of USP15—as observed in AML samples—would correlate with increased KEAP1 and decreased NRF2. In a majority of AML cells, we detected high levels of KEAP1 and low levels of NRF2 (Fig. 4D). In contrast, normal CD34+ cells express low levels of KEAP1 protein and high levels of NRF2 (Fig. 4D). We then quantified KEAP1 and USP15 protein expression, which revealed a significant positive correlation between KEAP1 and USP15 protein expression in AML cells (*P* = 0.016, *R*^2^ = 0.46) (Fig. 4E). These data support a model where the USP15-KEAP1-NRF2 redox signaling node is hijacked in AML cells.

### Inhibition of USP15 activates NRF2 and affects cellular ROS

**Fig. 5:**
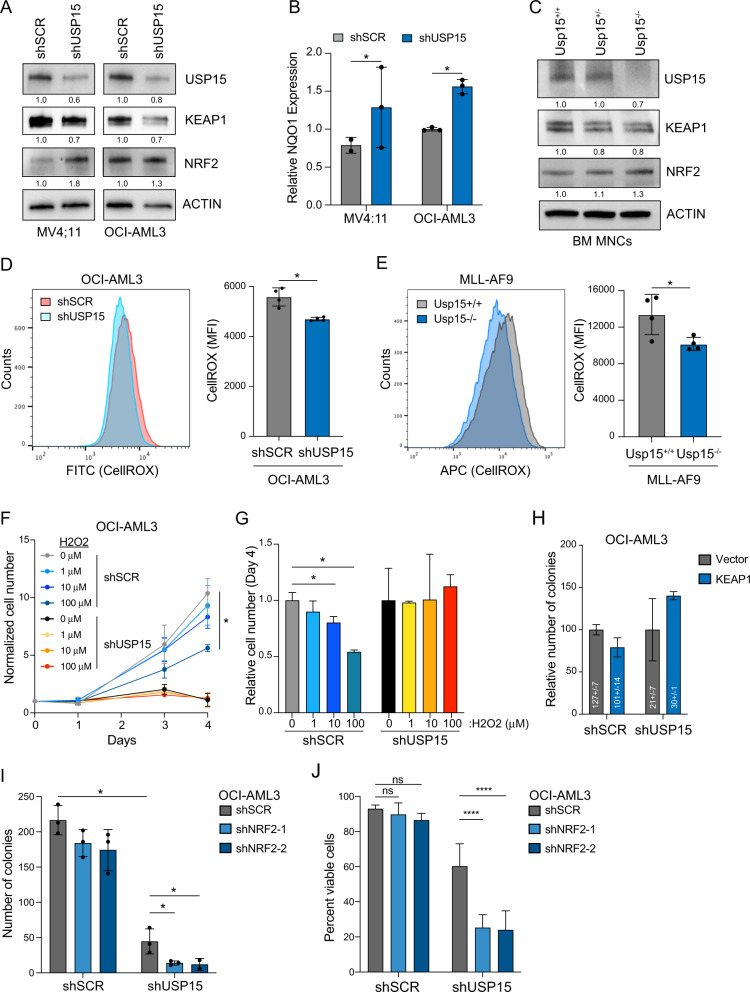
Inhibition of USP15 activates NRF2 and affects cellular ROS. **A** Immunoblot analysis of USP15, KEAP1, NRF2, and ACTIN in the indicated AML cells transduced with shRNAs targeting USP15 (shUSP15) or a scrambled control shRNA (shSCR). Values below blots represent the relative quantity of bands. Data reflects three independent assays. **B** Relative mRNA expression of the NRF2 target gene, NQO1, in the indicated AML cells transduced with shRNAs targeting USP15 (shUSP15) or a scrambled control shRNA (shSCR). Values were normalized to the shSCR controls for each (*n* = 3 replicates per group). **C** Immunoblot analysis of USP15, KEAP1, NRF2, and ACTIN in BM mononuclear cells isolated from Usp15+/+, Usp15+/−, and Usp15−/− mice. Data reflects three independent assays. **D** Left: a representative histogram of CellROX-Green intensity, indicative of cellular ROS, measured in OCI-AML3 cells transduced with shRNAs targeting USP15 (shUSP15) or a scrambled control shRNA (shSCR); right: bar graph of the median fluorescent intensity of CellROX (FITC) observed from OCI-AML3 cells transduced with shRNAs targeting USP15 (shUSP15) or a scrambled control shRNA (shSCR) (*n* = 4 per group). Data reflects two independent assays. **E** Left; a representative histogram of CellRox-Deep Red intensity, indicative of cellular ROS in Usp15+/+ and Usp15−/− cells expressing MLL-AF9; Right: summary of the median fluorescent intensity of CellROX-Deep Red, indicative of cellular ROS in MLL-AF9;Usp15+/+ and MLL-AF9;Usp15−/− AML cells (*n* = 4 per group). Representative data from 2 replicate experiments. **F** Cell growth of OCI-AML3 cells transduced with shRNAs targeting USP15 (shUSP15) or a scrambled control shRNA (shSCR) normalized to the live cells plated at Day 0. Cells were cultured in media supplemented with the indicated concentration of hydrogen peroxide (H2O2). Representative data from 2 replicate experiments (*n* = 3 for shSCR; *n* = 2 for shUSP15). **G** Summary of the live cell number on day 4 of OCI-AML3 cells expressing shRNAs targeting USP15 (shUSP15) or a scrambled control shRNA (shSCR) treated with the indicated concentration of H202. The relative cell number was normalized to the cells treated with vehicle control (water; 0 μM). *P < 0.05 (*n* = 3 per group). **H** Number of colonies from OCI-AML3 lines expressing empty-vector or overexpressing KEAP1, with and without knockdown of USP15. Colony counts were normalized to the vector controls within each shRNA group. Numbers within the histogram show actual colony numbers for each group (*n* = 3 per group). **I** Total number of colonies formed by OCI-AML3 cell lines expressing shRNAs targeting USP15 (shUSP15) or a scrambled control shRNA (shSCR) that were subsequently transduced with shRNAs targeting NRF2. *P < 0.05 (*n* = 3 per group). Data reflects two independent assays. **J** Cell viability of OCI-AML3 cells from panel (**I**) at the termination of a 4-day growth curve in liquid culture. ****P < 0.0001; two-way ANOVA. (*n* = 4 per group).

Next, to evaluate the functional role of USP15 in this redox pathway in AML cells, we knocked down or deleted USP15 and assayed the effects on KEAP1-NRF2 signaling and cellular ROS in AML cells. In MV4;11 and OCI-AML3 cells, knockdown of USP15 coincided with reduced protein expression of KEAP1 and a corresponding increase in NRF2 protein expression (Fig. 5A). The increase of NRF2 protein expression was more modest in shUSP15-OCI-AML3 cells relative to shUSP15-MV4;11 following transduction with the shRNAs, as OCI-AML3 cells have a slightly higher basal level of NRF2 (Fig. 5A). NRF2 is a transcription factor that positively regulates the expression of NAD(P)H Quinone Dehydrogenase 1 (NQO1), an anti-oxidant gene that offers chemoprotection by detoxification of quinones, which are secondary metabolites and a source of cellular ROS [38]. As expected, knockdown of USP15 in MV4;11 and OCI-AML3 cells resulted in a significant increase in *NQO1* mRNA expression (*P* < 0.05) (Fig. 5B). In parallel, we also evaluated the USP15-KEAP1-NRF2 axis in BM cells isolated from Usp15+/+, Usp15+/−, and Usp15−/− mice. In this orthogonal approach, we observed that deletion of USP15 resulted in decreased protein expression of KEAP1 and a corresponding increase in NRF2 protein expression (Fig. 5C). These findings suggest that USP15-mediated protection of KEAP1 is sufficient to restrict NRF2 expression in order to maintain an aberrant redox state in AML.

Based on our observations that inhibiting USP15 engages the KEAP1-NRF2 redox node, we reasoned that this mechanism could lead to fluctuations in cellular ROS in leukemic cells. To test this, we transduced OCI-AML3 cells with shRNAs targeting USP15 (or scrambled control) and then analyzed cellular ROS using a cell-permeable dye, CellROX-Green. OCI-AML3 cells expressing shUSP15 exhibited a significant reduction in the intensity of CellROX (FITC-positive cells) as compared to shSCR-OCI-AML3 cells (*P* < 0.05) (Fig. 5D), indicating that knockdown of USP15 engages the antioxidant response to lower levels of cellular ROS. To substantiate this observation, we extended this analysis to the USP15-deficient MLL-AF9 cells (described in Fig. 3A). Consistent with shUSP15-OCI-AML3 cells, Usp15−/−;MLL-AF9 cells exhibited a significant decrease in median fluorescent intensity as compared to Usp15+/+;MLL-AF9 cells (*P* < 0.05) (Fig. 5E). Based on our observations that AML cells generally exhibit high ROS and higher expression of USP15 and KEAP1 and corresponding suppression of NRF2, we hypothesized that further ROS induction would overwhelm the capacity of the leukemic progenitors to tolerate oxidative stress. While the knockdown of KEAP1 decreases cellular ROS, overexpression of KEAP1 induces endogenous ROS [39]. As expected, overexpression of KEAP1 resulted in increased cellular ROS (Fig. S3B, C). However, the concomitant knockdown of USP15 with KEAP1 overexpression limited the ROS increase in these AML cells (Fig. S3B, C). Similarly, we subjected Usp15-proficient and -deficient OCI-AML3 cells to an exogenous source of ROS and examined their viability and proliferation. Concordant with KEAP1 overexpression, administration of hydrogen peroxide suppressed AML cell growth in a dose-dependent manner (Fig. 5F, G). Although shUSP15-OCI-AML3 cells have diminished proliferation in vitro as compared to control cells, the addition of hydrogen peroxide to the cultures did not further impact their proliferative capacity (Fig. 5F, G). We next determined whether diminished expression of KEAP1 upon USP15 knockdown in AML cells contributes to the observed leukemic cell defects. Restoration of KEAP1 expression in shUSP15-OCI-AML3 cells resulted in a partial rescue of leukemic progenitor function as compared to control shUSP15-OCI-AML3 cells (Fig. 5H), suggesting that functional defect of AML cells upon loss of USP15 is in part mediated by the disrupted stoichiometry of KEAP1 in the redox response. Taken together these findings indicate that USP15 and KEAP1 maintain aberrant, high levels of cellular ROS in AML and that suppression of USP15 permits an antioxidant response to endogenous and exogenous sources of redox stress.

At steady-state, we observe that USP15-expressing AML progenitor cells persist with high cellular ROS (Fig. 5D, E) and concomitant suppression of the redox responses (Fig. 4C). Furthermore, knockdown of USP15 concomitantly impaired leukemic progenitor function and re-sensitized the cellular antioxidant response in these cells. We, therefore, reasoned that the observed activation of NRF2 in USP15-deficient leukemic cells provides a cytoprotective effect. To test this, we expressed shRNAs targeting NRF2 (shNRF2) in shUSP15-OCI-AML3 or shSCR-OCI-AML3 cells (Fig. S3D, E). The shSCR-OCI-AML3 cells alone were impervious to the knockdown of NRF2 (Figs. 5I, J and S3F, G). In contrast, combined knockdown of USP15 and NRF2 significantly impaired the cell viability and leukemic progenitor function of the AML cells (shNRF2;shUSP15-OCI-AML3 vs shSCR;shUSP15-OCI-AML3) (Figs. 5I, J and S3F). These findings suggest that AML cells rely on broken redox sensing, instigated by USP15-KEAP1 repression of NRF2-mediated antioxidant programming. Upon suppression of USP15, the KEAP1-NRF2 node is restored towards physiological levels in these AML cells, and the leukemic progenitor function is diminished as they become dependent on NRF2 for survival. Collectively, our data support a model in which the loss of USP15 in leukemic progenitor cells restricts leukemic cell growth and disrupts a critical node of redox homeostasis through KEAP1 and NRF2.

### A USP15 inhibitor impairs AML without affecting normal CD34+ cells in vitro

**Fig. 6:**
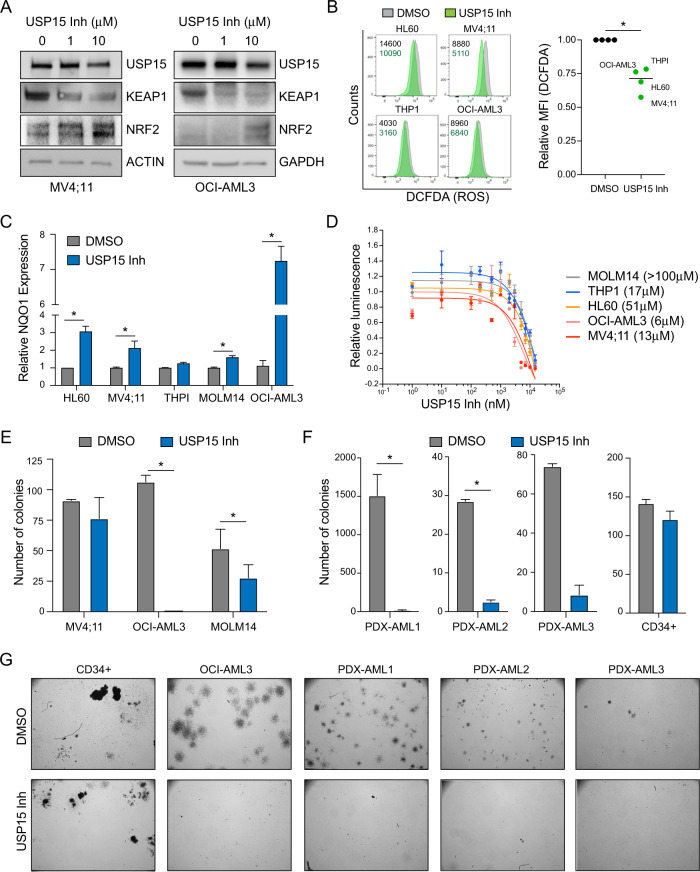
Inhibition of USP15 with a small molecule induces NRF2-medidated redox stress and impairs leukemic progenitors in vitro. **A** Immunoblot analysis of USP15, KEAP1, NRF2, and ACTIN/GAPDH in MV4;11 and OCI-AML3 cells treated with vehicle (DMSO, 0 μM) or the indicated concentrations of USP15 inhibitor for 24 h. Data reflects three independent assays. **B** Left; histogram flow plots of DCFDA analysis of cellular ROS in the indicated AML cells treated for 24 h with DMSO (gray) or 10 μM of USP15 inhibitor (green) with the raw MFI values set shown. Right; the normalized mean-fluorescent intensity of DCFDA in the AML cell lines versus their DMSO controls. A parametric, ratio-paired *t* test was performed. *P = 0.01. The centerline depicts the median. Data is from four independent cell lines. **C** Relative NQO1 expression in the indicated AML cell lines treated with vehicle (DMSO) or USP15 inhibitor (10 μM) for 24 h (*n* = 3 replicates). **D** Cell-Titer Glo relative luminescence was measured at 72 h with USP15 inhibitor in the indicated AML cell lines. Luminescence detected in inhibitor-treated cells was normalized to the vehicle control cells treated with DMSO (*n* = 3 replicates per dose). The IC50 values for each cell line are shown. **E** Total number of colonies formed by MV4;11, OCI-AML3, and MOLM14 cell lines in methylcellulose after 7 days of treatment with the USP15 inhibitor (10 μM). (*n* = 3 per group). Data reflects two independent assays. **F** The total number of colonies formed by three independent patient-derived-xenografted (PDX) AML samples and normal CD34+ cells after 7–10 days of treatment with the USP15 inhibitor (10 μM). *P < 0.05. **G** Representative colony images from healthy CD34+, OCI-AML3 leukemic cell line, and three PDX AML samples treated with DMSO or USP15 inhibitor.

DUB inhibitors represent an exciting emerging class of small molecules that are informative chemical biology tools and of great interest therapeutically. Using a pre-clinical small-molecule inhibitor of USP15 (USP15-Inh), we evaluated whether pharmacologically inhibiting USP15 DUB activity is a tractable therapeutic approach in AML cells. First, OCI-AML3 and MV4:11 cells treated with USP15-Inh were evaluated for KEAP1 and NRF2 protein expression by immunoblotting. Consistent with genetic deletion of USP15, inhibiting USP15 function with the USP15-Inh resulted in reduced KEAP1 expression and a corresponding increase in NRF2 (Fig. 6A). To test whether USP15-Inh can also reduce cellular ROS in leukemic cells, we treated a panel of AML cell lines with USP15-Inh for 24 h and evaluated cellular ROS using 2′,7′-dichlorofluorescin diacetate (DCFDA). In parallel, we measured mitochondrial ROS using the Mitosox stain, which is a biproduct of mitochondrial respiration and an indicator of mitochondrial oxidative stress. An increase in mitoROS can also be observed during a salvage mechanism to alleviate reductive states [40]. Constitutively low levels of KEAP1 induce reductive stress and restrict the regenerative capacity of HSPC function [41]. Consistent with our prior observations, AML cells treated with USP15-Inh resulted in reduced cellular ROS (Fig. 6B) and an increase in mitochondrial ROS (Fig. S4A, B) as compared to vehicle-treated cells. We also observed increased expression of the NRF2 target gene, NQO1, in AML cell lines treated with USP15-Inh as compared to vehicle-treated cells (Fig. 6C). Collectively, these data indicate that USP15 catalytic activity is important for restricting NRF2 through KEAP1, and its inhibition results in reduced cellular ROS in leukemic cells.

We next evaluated the effects of USP15-Inh on the viability of AML cell lines. OCI-AML3 cells (6 μM), MV4;11 (13 μM), and THP1 (17 μM) were the most sensitive to USP15-Inh, while HL60 (51 μM) and MOLM14 (>100 μM) were the least sensitive to USP15-Inh (Fig. 6D). Although all of the evaluated AML cell lines induced NQO1 expression upon USP15-Inh treatment, they were not all correspondingly sensitive to the effects of the inhibitor, suggesting that redox homeostasis regulation by USP15 varies across AML subtypes. We next evaluated the effects of USP15-Inh on the leukemic progenitor function of MV4;11, OCI-AML3, and MOLM14 cells. USP15-Inh suppressed leukemic progenitor function of OCI-AML3 cells, whereas MV4;11 and MOLM14 were generally less sensitive (Fig. 6E). To determine whether primary AML samples respond to inhibition of USP15, we treated 3 patient-derived xenograft (PDX) AML samples with USP15-Inh (10 μM) and evaluated their colony-forming ability in vitro. The PDX AML samples were all sensitive to USP15 inhibition as the leukemic progenitor function was reduced by greater than 90% for all samples treated with USP15-Inh relative to control cells (Fig. 6F, G). In contrast, healthy CD34+ hematopoietic progenitor cells were largely unaffected by the USP15 inhibitor at the same concentrations (Fig. 6F, G). These findings suggest that AML cells are dependent on USP15 function while normal hematopoietic cells can tolerate inhibition of USP15.

Based on our observations linking USP15 to the KEAP1-NRF2-ROS axis in leukemic cells, our data support a model wherein healthy hematopoietic progenitor cells more effectively tolerate a reduction in cellular ROS induced by USP15 inhibition. Several studies have indicated that hematopoietic stem cells maintain low levels of ROS and this cell state is important for preventing aberrant HSC activation. This is consistent with our observation reported here that healthy CD34+ cells robustly express NRF2 protein and exhibit low levels of USP15 and KEAP1 (Fig. 4D). Therefore, we postulate that inhibition of USP15 spares normal hematopoietic progenitors based on their preferentially high levels of NRF2 and their phenotypically low ROS states, compared to leukemic progenitors. Interestingly, single-cell RNA sequencing of activated versus non-activated murine HSCs [42] demonstrates that HSC activation and its associated increase in cellular ROS result in robust USP15 expression (Fig. S4C), further highlighting the functional link between USP15-low and ROS-low cellular states. In sum, our studies indicate that leukemic progenitor cells are uniquely reliant on USP15, thus offering a conceptual basis and a therapeutic window for targeting USP15 in AML.

## Discussion

Based on our observations that USP15 expression is selectively higher in AML, we investigated the role of USP15 in various human and mouse AML models and in normal hematopoiesis. Our studies revealed that USP15 is a critical modulator of the cellular redox homeostasis in AML that may be subverted for therapeutic benefit. Previous studies had identified USP15 as a deubiquitinase of KEAP1 [28, 29, 43]. KEAP1, a substrate adapter protein of E3 Cullin ligases, serves as the major negative regulator of the transcription factor NRF2 by controlling its stability and restricting its access to the nucleus [31]. Through its ability to engage antioxidant programs that confer tolerance to oxidative stress, the upregulation of NRF2 has been observed in several cancer models and has been implicated in chemoresistance [44–47]. However, our studies reveal a dysfunctional redox sensing mechanism in AML cells that is driven by high levels of USP15. We find that in leukemic progenitors, NRF2 expression is restricted compared to normal hematopoietic cells, despite their ROS-high state. Depletion of USP15 lowers levels of KEAP1 and activates NRF2, thus restoring this redox axis to normal physiological levels and modulating cellular ROS. Despite the upregulation of NRF2 under USP15-depleted conditions, leukemic cell function was overtly impaired in vitro and in vivo. When we depleted USP15 in AML samples compared to control cells, we observed activation of NRF2, a concomitant reduction in cellular ROS, and restricted leukemic progenitor function. We then reasoned that this activation of NRF2, driven in part by lower levels of KEAP1, and the coincident reduction in ROS indicated restoration of the redox-sensing mechanism. Expression of KEAP1 in USP15-depleted cells partially rescued leukemic progenitor function, indicating that leukemic progenitor function partly relies on an excess of KEAP1 that is enforced through USP15 activity. However, in USP15-depleted cells where KEAP1 remains low, survival of the dysfunctional leukemic progenitors becomes dependent on the cytoprotective effects of the NRF2. This is supported by our observations that concomitant knockdown of NRF2 and USP15 potently impairs leukemic progenitor function and cell viability. Taken together, this suggests that depletion of USP15 allows ROS sensing by KEAP1-NRF2, rendering the USP15-depleted cells dependent on NRF2 antioxidant programming. Importantly, USP15 was recently reported to have additional relevant functions in hematopoietic and chronic myelogenous leukemia models, including the regulation of the DNA damage response and in the suppression of p53 via deubiquitination of MDM2 [26, 32, 48]. Therefore, under USP15-depleted conditions, it is possible that compromised genome integrity and aberrant p53 activation induce a critical reliance on NRF2 activation and therefore a reduced redox state, for cell survival. However, this restoration of the KEAP1-NRF2 axis alone appears insufficient to overcome the barrier to leukemic progenitor function imparted by targeting USP15.

Cellular ROS had previously been considered categorically damaging, however recent studies, particularly in leukemia models, have demonstrated that cancer cells may subvert cellular ROS as secondary messengers that activate oncogenic transcriptional programs favoring cell survival in spite of this damaging redox state [10, 11]. Concordantly, it has been reported that AML progenitor cells intrinsically harbor high levels of cellular ROS, which supports their function through mechanisms that are increasingly understood. Our studies align well with this model based on our observations that USP15-high AML samples and experimental models exhibit increased progenitor function and higher levels of ROS compared to USP15-low leukemic cells which demonstrate decreased progenitor function and lower levels of ROS. In contrast, functionally characterized leukemic stem cells (LSCs) are a subset of leukemic cells defined by comparatively low levels of ROS (ROS-low), which confer increased quiescence and engraftment potential compared to the ROS-high LSCs [8]. Importantly, the ROS-low cell state sensitizes LSCs to genetic and pharmacologic perturbations in redox homeostasis [49, 50]. Our studies reported here do not evaluate LSC populations, however, our findings do support the model wherein upper and lower thresholds in redox homeostasis can be perturbed to target leukemic cells [51].

An important corollary to mechanisms regulating oxidative stress is the ability of a cell to sense and respond to reductive stress, defined partly by critically low levels of cellular ROS. These thresholds vary with the redox state of the cell, which is dependent on a delicate balance of metabolism, oxidative stress, antioxidant defenses in HSPCs [52]. For example, prolonged inhibition of KEAP1 and aberrantly sustained NRF2 activation have been shown to induce reductive stress [53], triggering a ubiquitin-dependent switch that preserves redox homeostasis by ramping up metabolism, which was recently reported in myoblasts [40]. Similarly, in USP15-low AML patient samples—which we now understand to be associated with a comparatively lower ROS state—we observe enrichment in RNA expressed from genes involved in mitochondrial translation, MYC targets, and tRNA biosynthesis (Fig. 4A). Moreover, we observe that this induces a dependence on NRF2 for cell survival. Our observations herein and the work of others raise the possibility that USP15 function is intertwined with metabolism and mitochondrial control [54–56] in hematopoietic cells, which we did not directly address in this study. An implication of these findings is that USP15-low leukemic cell populations may be further targeted by impinging on this oxidative-reductive stress response. Specifically, since we observed that NRF2 is required for the maintenance of leukemic cell function under a USP15-low cell state, targeting NRF2 in USP15-low cells may also be an attractive therapeutic approach in this context. However, the dichotomy of high ROS levels and increased leukemic cell function is reversed within immunophenotypically defined LSCs. ROS-low-defined subsets of disease-propagating LSCs exhibit increased quiescence, stem cell function, and substantially increased levels of pro-survival BCL-2, on which they are dependent for their survival and resistance to chemotherapy [8]. Importantly, this dependency results in acute, potent sensitivity to the combination treatment of AML with azacytidine and BCL-2 inhibitor Venetoclax [57]. In light of these crucial findings, our report here on USP15 indicates a biological basis for ROS-high and ROS-low cell states in bulk leukemic progenitor cells. We postulate that understanding these mechanisms in progenitor cells will allow us to identify approaches to lower cellular ROS and sensitize heterogeneous cell populations to this effective treatment strategy, potentially through pharmacological inhibition of USP15 alone or in combination with NRF2 inhibition. There is evidence to suggest that the combination of hypomethylating agents and Venetoclax elicits degradation of NRF2 [58]. While our studies herein do not directly address whether this USP15-dependent redox mechanism is active in LSCs or HSCs, by mining single-cell RNA-sequencing data, we observed an increase in USP15 transcripts in activated murine HSCs compared to non-activated counterparts (Fig. S4B), further delineating a correlation between high USP15 expression and ROS-high cell states. Future studies of USP15-dependent regulation of KEAP1 and NRF2 and their effects on redox homeostasis may also inform mechanisms of stem cell activation, which is requisite for successful stem cell transplantation. Allogeneic stem cell transplantation remains the only curative therapy for AML.

A major gap in our understanding of USP15 in AML is the lack of understanding of the regulation of USP15 expression in this context. One study in multiple myeloma models revealed that USP15 transcripts increase in response to LPS stimulation and NF-κB activation [59]. In a mechanistic study in HEK293 cells, USP15 protein translation was modestly increased with TGF-β stimulation and this effect could be mitigated by PI3K or mTOR inhibition [60]. An enticing premise for the observed high levels of USP15 would be that inflammatory signaling in malignant BM niches [61, 62] activates these pathways, thus driving USP15 expression and promoting cell survival in response to stress. It will be interesting to determine if constitutive overexpression of USP15 and inflammation in normal BM promotes enhanced progenitor function or stem-like properties.

Lastly, through the application of a pre-clinical small-molecule inhibitor, our data demonstrate that USP15 is an actionable drug target in AML cells. In congruence to our RNA-targeted and genetic models of USP15 depletion, treatment of leukemic cell lines and patient-derived xenograft samples with the USP15 inhibitor reproduced the downregulation of KEAP1 and increase in NRF2 and its target gene NQO1. Altogether based on our genetic knockout mouse model and on our observations that the USP15 inhibitor bore no immediate effect on healthy CD34+ cells in vitro, this mechanism appears to be dispensable for normal hematopoietic cells. In fact, we observed that healthy CD34+ cells robustly express NRF2 protein and exhibit low levels of USP15 and KEAP1, compared to leukemic cells, indicating that the redox mechanism induced by USP15-inhibition is already intact in these cells. Taken together, our findings describe a role for USP15 in leukemic cell redox biology and identify an actionable therapeutic window for targeting USP15 in AML models.

## Materials and methods

### Lead contact and materials availability

Further information and requests for resources and reagents should be directed to and will be fulfilled by the Lead Contact, Daniel Starczynowski (daniel.starczynowski@cchmc.org). All unique resources generated in this study are available from the Lead Contact with a completed Materials Transfer Agreement.

### Experimental model and subject details

#### Animals

This study utilized murine animal models, consisting of adult mice aged 1–6 months. Usp15−/− mice were generated by CRISPR/Cas9-mediated targeting of conserved USP15 exon 2 using embryonic microinjection of C57Bl/6 wildtype mice, as previously described [26]. For total and competitive BM transplantation studies, a mixture of male and female wildtype BoyJ mice was used as recipients, and mice were randomly assigned to experimental groups. Congenic BoyJ recipients were conditioned with lethal, 10 Gy total body irradiation prior to all BM transplantations. All transplantations were performed using BM mononuclear cells from C57Bl/6 donors unless specified otherwise. For xenotransplantation studies, human AML cells (OCI-AML3) were tail-vein injected into pre-conditioned recipients of the NSGS immune-compromised strain. NSGS mice received sublethal irradiation of 250 cGy to promote engraftment of the human tissue. Animals were bred and housed in the Association for Assessment and Accreditation of Laboratory Animal Care-accredited animal facility of Cincinnati Children’s Hospital Medical Center. All experiments conform to the regulatory standards of the Institutional Animal Care and Use Committee (IACUC) and adhere to IACUC-approved protocols.

#### Human samples

BM samples from patients with AML were obtained with written informed consent and approved by the institutional review board of Cincinnati Children’s Hospital Medical Center. These samples had been obtained within the framework of routine diagnostic BM aspirations after written informed consent in accordance with the Declaration of Helsinki. Human hematopoietic stem/progenitor cells from normal donor populations were enriched for CD34+ by the Cell Processing Core at Cincinnati Children’s Hospital Medical Center. PDX-AML1 (Av418-L) contains a complex karyotype with PTPN11 and TP53 mutations. PDX-AML2 (225-16-B) contains a CBFA2T3/GLIS2 translocation. PDX-AML3 (Av226-L) contains a complex karyotype with CDKN2A/B deletion. No other known mutations were reported. All cells were maintained in culture with IMDM supplemented with 10% FBS, 20 ng/mL human IL-6, 20 ng/mL human IL-3, and 100 ng/mL human SCF.

#### Cell lines

All cells were cultured at 37 °C with 5% carbon dioxide. All cell lines were directly purchased from or have been authenticated by STR Profiling Services from ATCC (ATCC 135-XV-10), including MOLM13, MOLM14, MV4;11, MOLT16, HL60, OCI-AML2, OCI-AML3, THP1, SET2, and F36P. MDS-L cells were gifted by the laboratory of Dr. Kaoru Tohyama and previously described [63–65]. MOLM13, MOLM14, MV4;11, MOLT16, HL60, THP1, and F36P cells are cultured in RPMI supplemented with 10% fetal bovine serum and 1% penicillin/streptomycin. OCI-AML2, OCI-AML3, and SET2 were cultured in RPMI supplemented with 20% fetal bovine serum and 1% penicillin/streptomycin. MDSL cells were cultured in RPMI supplemented with 10% fetal bovine serum, 1% penicillin/streptomycin, and 10 ng/mL of human interleukin (IL)-3.

### Method details

#### Cell lines and cell culture

OCI-AML3 cells were cultured in alpha-MEM with 20% FBS and 1% penicillin/streptomycin. MV4;11 cells were cultured in RPMI with 10% fetal bovine serum and 1% penicillin/streptomycin. During viral infection with MLL-AF9-GFP encoding retrovirus and following CFC expansion, murine HSPCs were cultured in IMDM with 10% FBS and 1% penicillin/streptomycin and 10 ng/mL of each murine IL-3, human IL-6, and murine SCF.

#### Viral constructs, transduction, cell sorting, and BM transplantation

To generate a USP15-deficient MLL-AF9 model, USP15+/+and USP15−/− lineage negative (Lin−) BM cells from C57Bl/6 mice were transduced with retrovirus encoding MLL-AF9 and a GFP reporter [37]. Due to the low transduction efficiency of these cells, three rounds of colony replating were performed to enrich for MLL-AF9-expressing AML cells. By the third plating, we confirmed that nearly all colonies express MLL-AF9 as determined by FACS (>95% GFP+ cells). Equal numbers of Usp15+/+ and Usp15−/− MLL-AF9-expressing (GFP+) AML cells were then transplanted into lethally irradiated recipient BoyJ mice: 500,000 GFP + Usp15+/+ or Usp15−/− MLL-AF9-expressing cells and 200,000 helper BM cells per mouse. Knockdown of USP15 in human AML cell lines and in CD34+ cells was performed with lentivirus encoding PLKO.1-shUSP15-2-mCherry, PLKO.1-shUSP15-4-mCherry, with scrambled-shRNA controls for each construct as previously described [66]. All cells were sorted using FACS Aria equipment or Sony SH800S under BSL2 protocol. OCI-AML3 cell lines stably expressing control or NRF2-targeted shRNA were generated by delivering lentivirus encoding MISSION-shRNA constructs PLKO.1-shNRF2-1-puroR, PLKO.1-shNRF2-2-puroR, or PLKO.1-shScramble-puroR. Cells were selected in culture for 3 days with 0.5 ug/mL puromycin and partial knockdown of NRF2 was confirmed by western blot.

#### Western blotting

Primary antibody dilutions were prepared at 1:1000 to 1:500 in 5% BSA or dry milk in TBS with 0.05% v/v Tween. Protein samples prepared directly from lysate were prepped in 5X SDS sample buffer. Westerns were run in BioRad equipment in SDS buffer, using BioRad gradient gels, at 175 V for 42 min. Precision plus dual color protein marker (BioRad) was used in parallel to samples. Proteins were wet-transferred onto nitrocellulose membranes (0.2 µM, BioRad) at 100 V for 1 h. Membranes were blocked in 5% BSA or milk for 1 h RT depending on antibodies to be used. Membranes were incubated with primary antibodies overnight at 4° and washed for a total of 30 min in TBST before adding secondary diluted to 1:10,000 through 1:5,000 in 5% milk. Antibodies used in immunoblots are as follow: Anti-USP15 (Proteintech 14354-1-AP) used at 1:500-1:1000, Anti-Vinculin E1E9V XP (Cell Signaling Technologies #13901) used at 1:1000, Anti-KEAP1 P586 (Cell Signaling Technologies #4678) used at 1:500, Anti-NRF2 (Cell Signaling Technologies #12721) used at 1:500, anti-GAPDH D16H11 XP (Cell Signaling Technologies #5174) used at 1:1000, anti-pan-Actin (Cell Signaling Technologies #4968) used at 1:1000.

#### RNA expression analyses

Prior to performing qPCR to detect levels of mRNA in samples, RNA was isolated using the Zymo Quick-RNA kit. All samples are normalized to an internal control probe, Actin, and performed in a minimum of triplicates.

Taqman probes detecting USP15 and NQO1 were prepared in 2X Taqman Master Mix with 1–2 µL of cDNA added per replicate depending on the concentration of RNA in each experiment.

#### BM extraction, HSPC enrichment, and colony assays

To generate BM mononuclear cells for immunoblots, six murine hind-limb bones were collected, crushed, and filtered in PBS [67]. BM cells were pelleted and subjected to red-blood-cell lysis buffer to isolate BM mononuclear cells. For colony-forming assays involving cKit+ Usp15+/+ and Usp15−/− BM cells, enrichment of HSPCs was performed by AutoMACS positive selection using CD117 murine microbeads for cKit+ enrichment. Prior to the generation of MLL-AF9-expressing murine HSPCs, the EasyStep Mouse Hematopoietic Progenitor Cell Enrichment Kit (Stem Cell Technologies 19856) was used for lineage depletion. Murine HSPCs were plated in 1 mL of MethoCult® GF M3434 (Stem Cell Technologies) per well of an untreated 6-well culture plate. Total colonies were counted automatically in 5–10-day intervals and confirmed manually, depending on the growth rate of the cells. Where colony images and counts are provided, images were captured using the StemVision plate reader (Stem Cell Technologies).

#### Detection of cellular ROS

For detection of cellular ROS, CellROX Green and CellROX Deep Red was utilized at a working concentration of 5 µM. Cultured cells were collected, spun down, and stained in 500 µL of warmed PBS containing the working concentration of dye. Cells were stained for 30 min at 37° protected from light. Cells were washed with 1X pre-warmed PBS and transferred to capped flow tubes and kept on ice during acquisition. Samples were read within one hour of the completion of the stain. For detection of mitochondrial superoxide species, MitoSOX Red was employed utilizing the same dilutions and method as described for CellROX reagents. CellROX green was collected on FITC, CellROX Deep Red on APC, and MitoSOX Red on PE. For samples that received USP15 inhibitor, cells were pre-treated with DMSO as vehicle control or 10 µM of USP15-inhibitor for 24 h in culture prior to ROS detection protocols. When DCFDA was used for the detection of ROS, cells were stained with 20 µM final concentration according to manufacturer-provided protocol. Samples were analyzed on BD FACS Canto machines and data were analyzed using FlowJo software.

#### USP15 inhibitor

The USP15 inhibitor used in this study was provided by Forma Therapeutics.

### Quantification and statistical analysis

#### Gene-set enrichment analysis

Gene set enrichment analysis (GSEA, https://www.gsea-msigdb.org/gsea/) was performed using the BEAT-AML dataset. The AML patient data were divided into two groups, the top 25% and bottom 25% USP15-expressing samples (USP15-high and USP15-low respectively) depending on the z-score metric (cutoff: |*z*-score| > 1.2). Enriched gene sets were searched against the C2 gene set collection from MSigDB (http://www.gsea-msigdb.org/gsea/msigdb/) and enrichment results were permuted 1000 times using the gene-set permutation option. Default values were used for all other parameters.

#### Immunoblot image quantification

Protein quantities were determined using the gel analysis features in ImageJ software. Background subtraction was performed, lanes were set, and the relative mean gray area values for each protein were calculated by plotting all lanes and using the Label Peaks function. Subsequently, each band was normalized to its internal loading control value in Microsoft Excel to determine the relative protein expression across samples. Finally, all relative expression values for a given protein were normalized to the vehicle- or shRNA-experimental control samples.

#### Statistical analysis

To directly compare two experimental groups, unpaired nonparametric *t*-tests were used to calculate *P*-values unless otherwise specified. For experiments with more than two conditions within a group, two-way ANOVA was applied unless otherwise specified. Statistical significance was determined if *P* < 0.05 and indicated with asterisks. N.s. or absence of asterisk indicates not statistically significant. Error bars depict the mean ± standard deviation. Information on biological and technical replicates, “*n*”, for each experiment can be found in the corresponding Figure Legend. All experiments were either repeated twice minimum in the laboratory or validated by the corresponding orthogonal assays and model systems shown here. Sample sizes were chosen based on the degree of variability observed in similar experiments and transplantation experiments were performed to detect relative treatment differences of 65% with 80% power at a significance level of 0.05. Inclusion/exclusion criteria were not applied, there was no blinding, and recipient animals were allocated to treatment groups randomly at the time of transplantation. All graphs and analyses were generated using GraphPad Prism software.

## Supplementary information

Supplemental Figures 1–4
