# Supplementary material for: The deubiquitinase USP15 modulates cellular redox and is a therapeutic target in acute myeloid leukemia
Source: Leukemia. 2021 Aug 31;36(2):438–51. doi: 10.1038/s41375-021-01394-z (PMC8807387; doi:10.1038/s41375-021-01394-z)
Supplement: Supplementary file 1 — Supplemental Figures 1–4 [file 41375_2021_1394_MOESM1_ESM.pdf]

Supplemental Figure 1

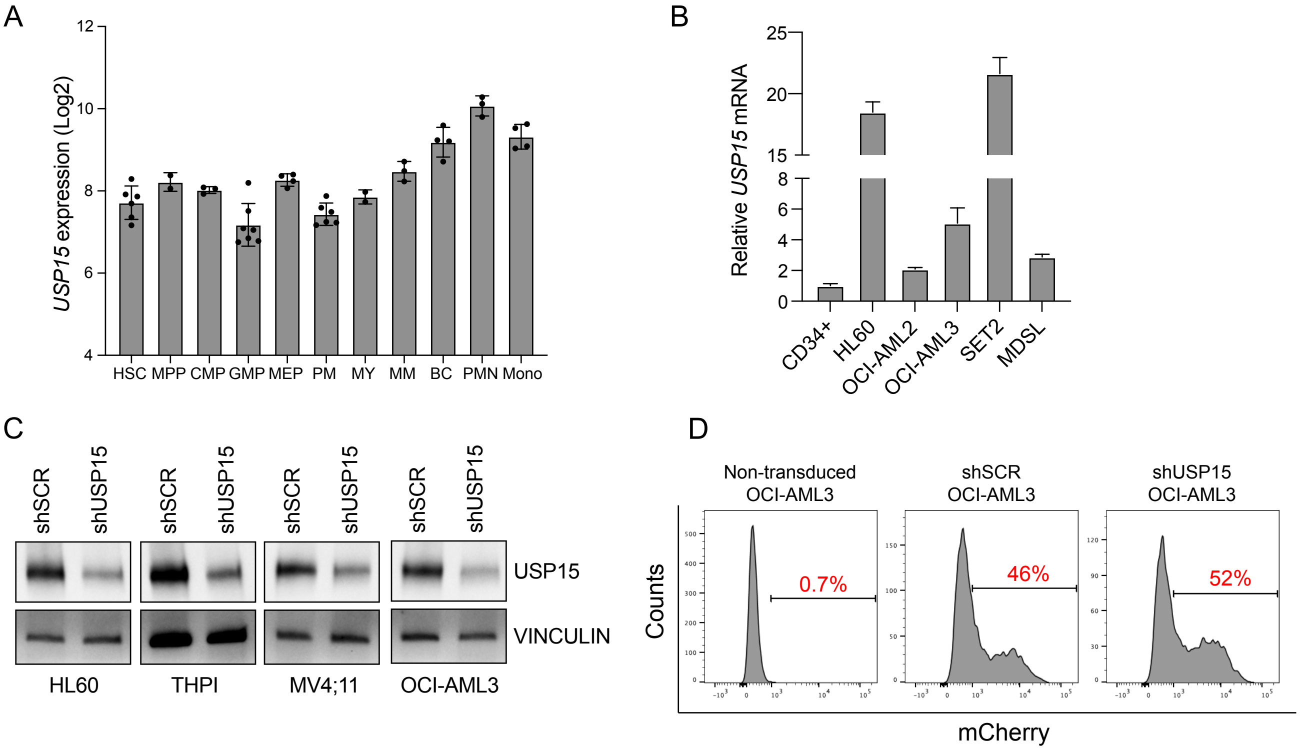

Figure S1. Expression of USP15 mRNA and knockdown human AML cell lines. (**A**) Median USP15 RNA expression in normal hematopoietic cell populations determined by RNA-sequencing obtained from the BloodPool study. (**B**) Relative USP15 mRNA in normal CD34+ cells and leukemic cell lines, determined by qPCR and normalized to Actin in each cell type. (**C**) Immunoblot blot of USP15 in the indicated AML cell lines expressing shRNAs targeting USP15 (shUSP15-mCherry) or a scrambled control shRNA (shSCR-mCherry). (**D**) FACS histogram plots indicating percent mCherry+ fractions in the OCI-AML3 cells transduced with shSCR-mCherry or shUSP15-mCherry prior to xenograft.

Supplemental Figure 2

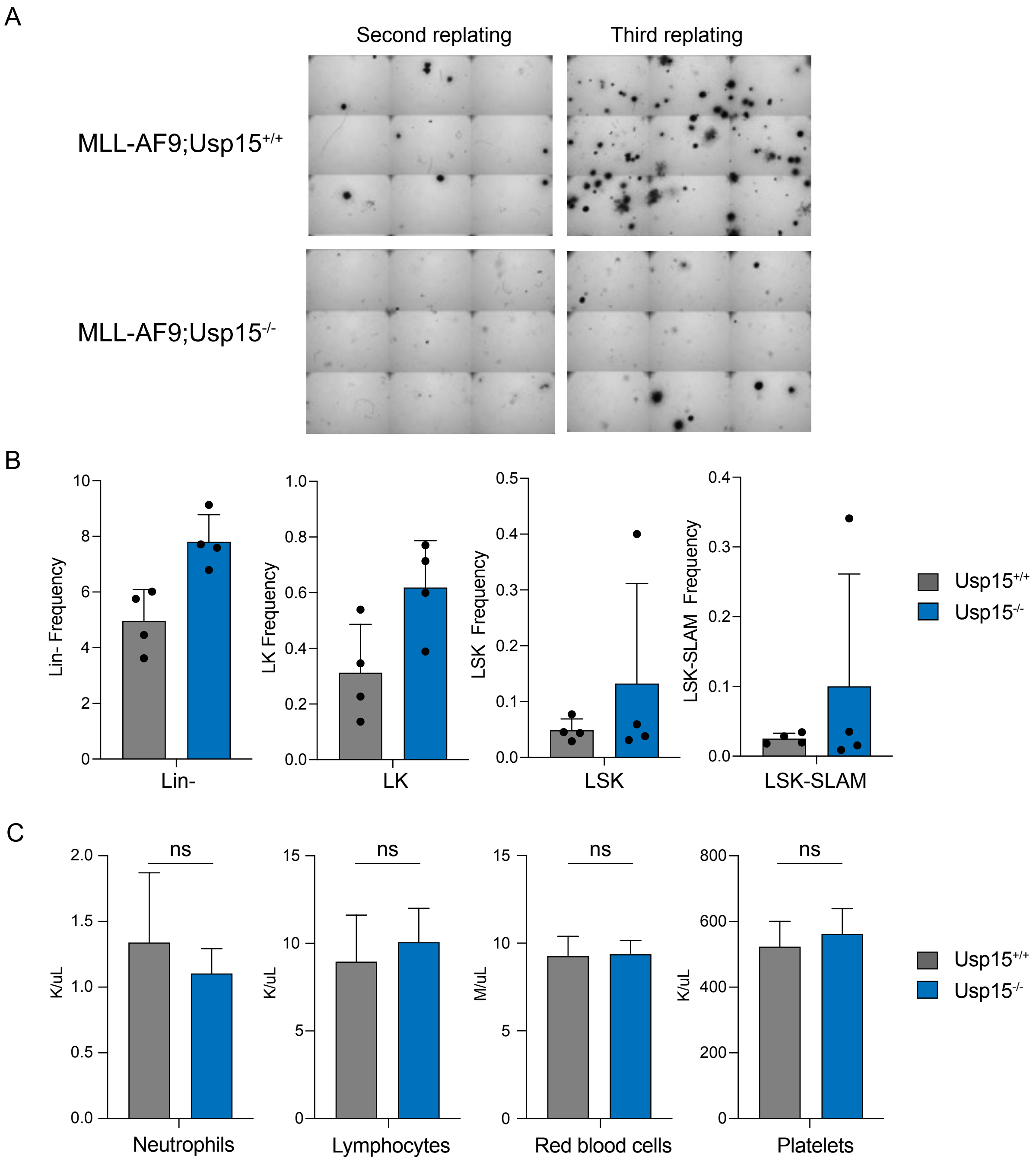

Figure S2. Generation of MLL-AF9 AML and analysis of mice engrafted with USP15-/- BM cells. (**A**) Images of colony-forming cell assays taken at the second and third serial replatings of Usp15+/+ and Usp15-/- Lin- BM cells that were transduced with MLL-AF9. (**B**) Proportions of Lineage-negative (Lin-), Lin-;c-Kit+ (LK), Lin-;c-kit+;Sca1+ (LSK), and LSK;CD48-;CD150+ (LSK-SLAM) cells in the BM of mice engrafted with Usp15+/+ and Usp15-/- CD45.2+ BM cells >1 year post-transplant (n = 4). (**C**) Complete blood counts of peripheral blood collected from mice engrafted with Usp15+/+ and Usp15-/- BM cells 5 months post-transplantation (Usp15+/+ n=15; Usp15-/ n=14). ns, not significant.

Supplemental Figure 3

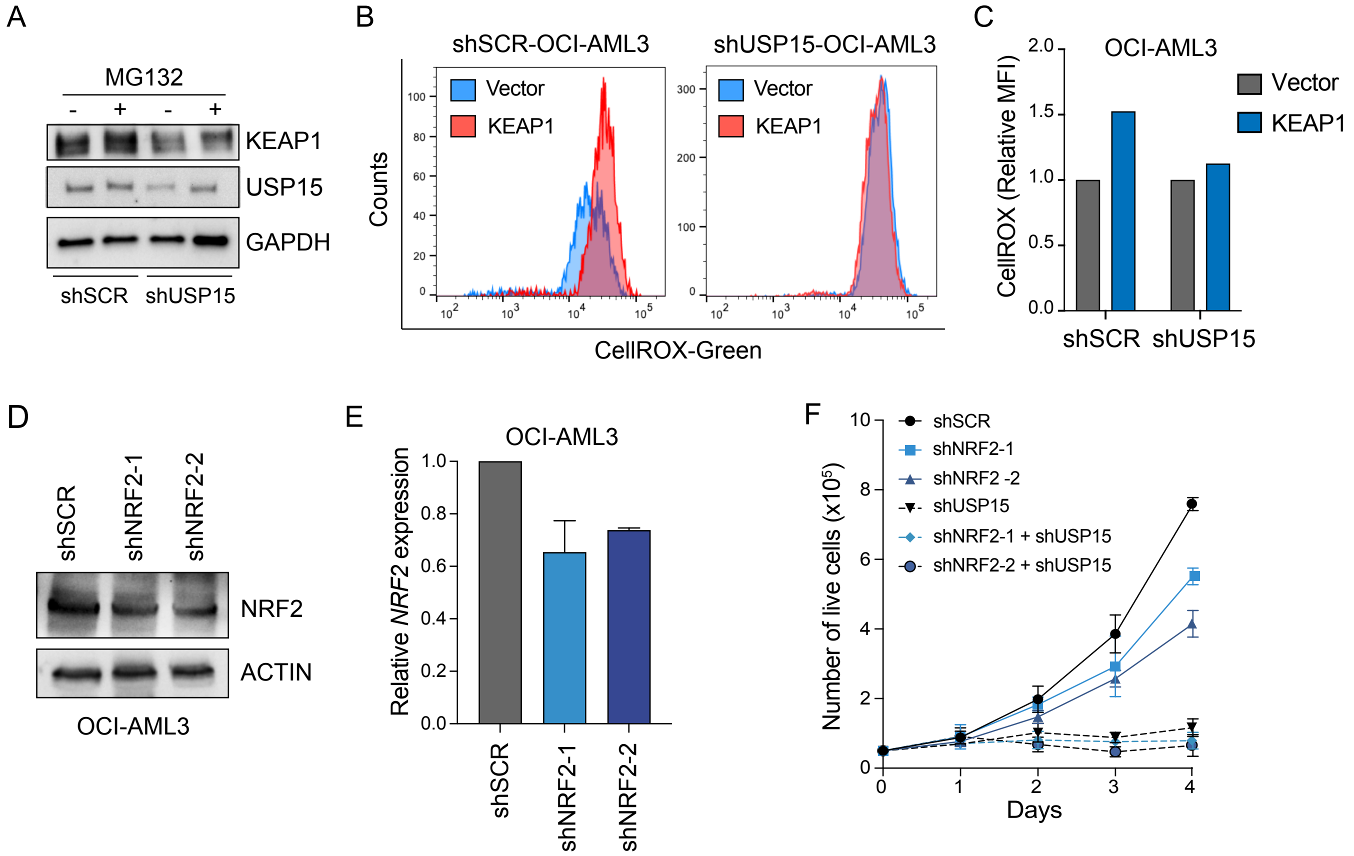

Figure S3. Knockdown of USP15 and NRF2 in a human AML cell line. (**A**) Western blot of KEAP1, USP15, and GAPDH in OCI-AML3-shSCR or OCI-AML3-shUSP15 cells treated with vehicle or 25uM of MG132 for 5 hours. (**B**) Histogram plot of CellROX-Green intensity for detection of cellular ROS in OCI-AML3 lines expressing empty-vector or overexpressing KEAP1, with and without knockdown of USP15. Shown is a representative experiment. (**C**) Bar graph of relative mean-fluorescent intensity of KEAP1-overexpressing cells from panel A, normalized to their vector-controls. (**D**) Immunoblot of NRF2 in OCI-AML3 cells expressing two independent shRNAs (co-expressing a puromycin resistance gene) targeting NRF2 (shNRF2) or a scrambled control shRNA (shSCR). Transduced cells were selected with puromycin for >3 days. The selected cells were subsequently transduced with shRNAs (co-expressing mCherry) targeting USP15 (shUSP15) or a scrambled control shRNA (shSCR) (for Figure 5I, J).(**E**) Relative mRNA expression of *NRF2* in puro-selected OCI-AMl3 cells, determined by qPCR. (**F**) Cell growth curve of OCI-AML3-shSCR or OCI-AML3-shUSP15 cells expressing puro-CTRL shRNA (CTRL) or shNRF2.

Supplemental Figure 4

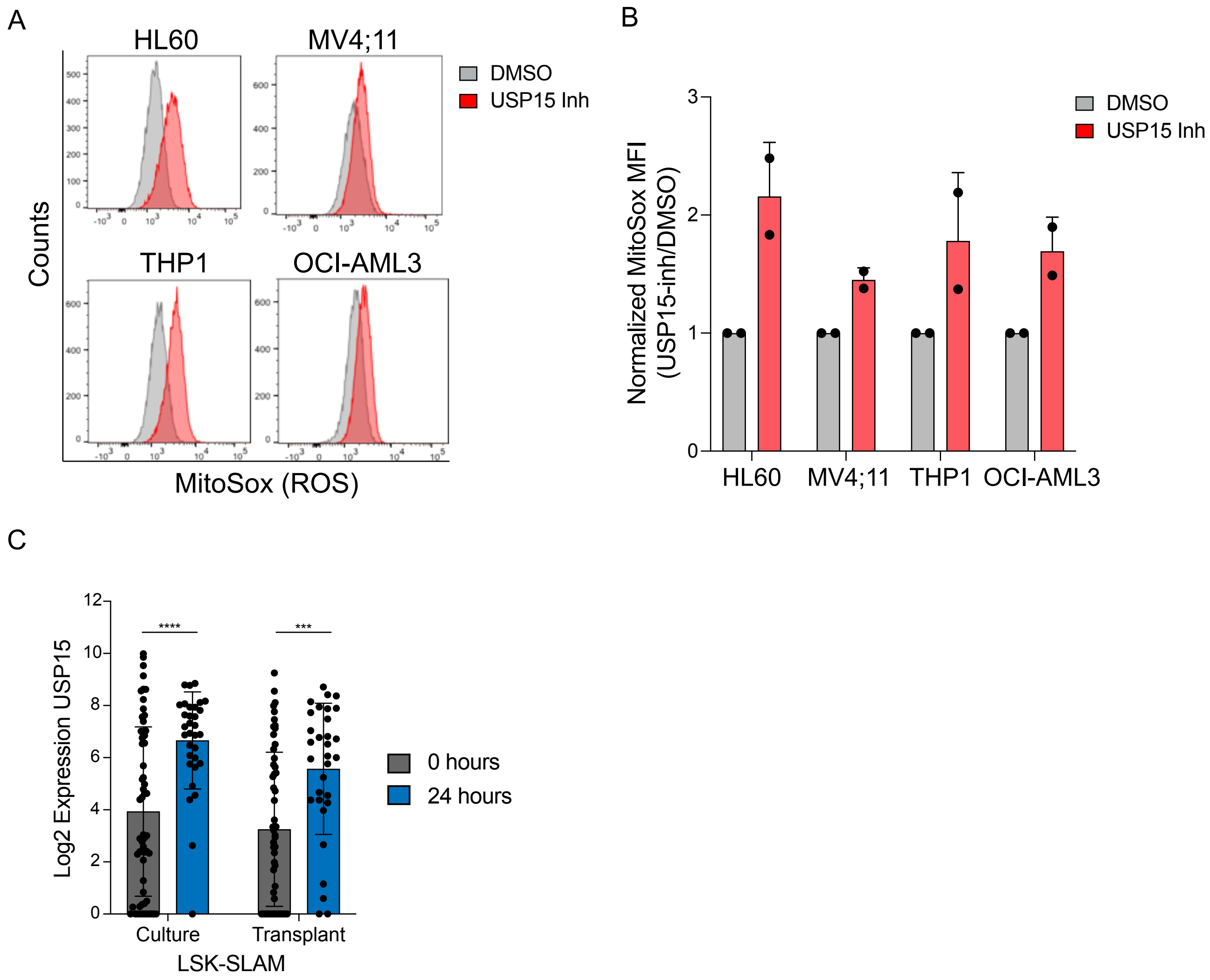

Figure S4. Mitochondrial reactive oxygen species in AML cells treated with USP15 inhibitors and expression of USP15 in stressed hematopoietic cells. (**A**) Histogram flow plots of MitoSox analysis for the detection of mitochondrial superoxide in the indicated AML cells treated for 24 hours with DMSO (grey) or 10 uM of USP15 inhibitor (Red). (**B**) Normalization of MitoSox MFI in cells treated in A. Two independent experiments, n=2. (**C**) Single-cell RNA-sequencing reads (Log2) for USP15 in murine LSK-SLAM cells that were isolated from mice and either placed into culture or immediately transplanted. RNA-sequencing was performed at 0 hours (grey), directly after each treatment, or 24 hours after treatment as the LT-HSCs activated (blue). ****, $P < 0.00005$.
